# Facile synthesis of bio-based nitrogen- and oxygen-doped porous carbon derived from cotton for supercapacitors

**DOI:** 10.1039/c7ra11475c

**Published:** 2018-01-22

**Authors:** Lan Zhang, Lu Xu, Yagang Zhang, Xin Zhou, Letao Zhang, Akram Yasin, Lulu Wang, Keke Zhi

**Affiliations:** Xinjiang Technical Institute of Physics and Chemistry, Chinese Academy of Sciences Urumqi 830011 China ygzhang@ms.xjb.ac.cn +86-991-3838957 +86-18129307169; University of Chinese Academy of Sciences Beijing 100049 China; Department of Chemical & Environmental Engineering, Xinjiang Institute of Engineering Urumqi 830023 China

## Abstract

Biomass-derived O- and N-doped porous carbon has become the most competitive supercapacitor electrode material because of its renewability and sustainability. We herein presented a facile approach to prepare O/N-doped porous carbon with cotton as the starting material. Absorbent cotton immersed in diammonium hydrogen phosphate (DAP) was activated at 800 °C (CDAP800s) and then was oxidized in a temperature range of 300–400 °C. The electrochemical capacitance of the impregnated cotton was significantly improved by doping with O and N, and the yield was improved from 13% to 38%. The sample oxidation at 350 °C (CDAP800-350) demonstrated superior electrical properties. CDAP800-350 showed the highest BET surface area (1022 m^2^ g^−1^) and a relatively high pore volume (0.53 cm^3^ g^−1^). In a three-electrode system, the CDAP800-350 electrodes had high specific capacitances of 292 F g^−1^ in 6 M KOH electrolyte at a current density of 0.5 A g^−1^. In the two-electrode system, CDAP800-350 electrode displayed a specific capacitance of 270 F g^−1^ at 0.5 A g^−1^ and 212 F g^−1^ at 10 A in KOH electrolyte. In addition, the CDAP800-350-based symmetric supercapacitor achieved a high stability with 87% of capacitance retained after 5000 cycles at 5 A g^−1^, as well as a high volumetric energy density (18 W h kg^−1^ at 250 W kg^−1^).

## Abstract 2

Biomass-derived O- and N-doped porous carbon has become one of the most competitive supercapacitor electrode material because of its renewability and sustainability.

## Graphical abstract

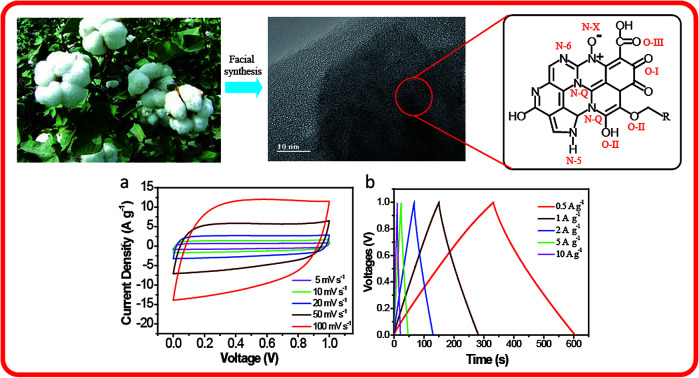

## 1. Introduction

The development of new materials for energy storage systems has attracted tremendous attention. With limited availability of fossils fuels, designing functional materials for sustainable and alternative energy resources is a feasible and promising approach.

Supercapacitors have been widely used in commercial and industrial equipment because of their higher power density, shorter charge–discharge times and longer lifetime than traditional energy storage devices.^1^ Carbon materials, such as carbon nanotubes, templated carbons, carbon spheres and graphene,^2–4^ are commonly studied. Carbon materials retain electrical charge by an electrostatic double layer and electrochemical pseudocapacitance.^5^ Generally speaking, porous carbon materials have superior electrochemical performance as supercapacitor electrodes due to their good chemical and physical stability, large specific surface area, high conductivity, and special hierarchical structure.^6–8^ Compared to the fossil-fuel based approach, the biomass-derived carbon materials display distinct advantages, such as low cost, availability and sustainability.^9^ From a practical application perspective, it is highly desirable to develop a facile approach rather than a complicated and tedious one.^10^ Notably, biomass-based carbon materials can be prepared by selecting proper raw materials; more and more research has been focused on finding biomass-derived materials such as cotton,^8^ wood sawdust,^11^ and orange peel^5^ as desirable precursors.

During the chemical activation process, different carbon precursors are mixed with chemical reagents such as ZnCl_2_,^12^ H_3_PO_4_,^13^ NaOH^14^ and KOH.^15^ For example, Chen *et al.*^16^ reported nitrogen-doped carbon by using melamine and carbonized at 800 °C. The as-prepared carbon showed a capacitance of 180 F g^−1^ at 0.5 A g^−1^. Tian *et al.*^15^ reported micropore-activated carbon as supercapacitors from cotton stalk *via* KOH chemical activation method, showing a specific capacitance of 254 F g^−1^ at a current density of 0.2 A g^−1^.

However, the disadvantages of using these corrosive chemicals are this type of process generates a large amount of wastewater during washing to remove the chemicals. Therefore, it is advantageous to develop a facile and green process with less corrosive and more benign chemical reagents. (NH_3_)_2_HPO_4_ (diammonium hydrogen phosphate, DAP) was found to be one of them because it is less corrosive after carbonization. Furthermore, heteroatom doping, such as oxygen, nitrogen, fluorine or phosphorus^17–19^ is an effective strategy to enhance the electrochemical performance of the porous carbon materials.^20^ Oxygen-containing groups can improve the surface wettability and facilitate more active sites for charge storage.^21^ The introduction of nitrogen doping could facilitate the mobility of negative charges on the carbon surfaces, thus improving capacitance.^22^

As one of the most important agricultural crops in the world, cotton contains abundant cellulose, hemicellulose and lignin. The annual cotton production is up to 2500 million tons. Herein, we reported a very facile, green pyrolysis strategy for preparing nitrogen- and oxygen-doped porous carbon for supercapacitor electrodes from bio-derived cotton resource. DAP was proved to be an effective activation reagent. Compared to other corrosive reagents, DAP was not only more eco-friendly and economical, but also significantly improved the final yield of porous carbon product (34%), which was improved by about 3-fold compared to that by unimpregnated cotton (13%). Furthermore, DAP was found to be an effective N-doped reagent and oxidation treatment helped introducing O, which facilitated better surface wettability and achieved larger specific surface area (1022 m^2^ g^−1^). The CDAP800-350 exhibited superior performance (292 F g^−1^ at 0.5 A g^−1^) than the controls both in three-electrode systems and two-electrode systems (270 F g^−1^, 0.5 A g^−1^), which was proposed to be because oxygen and nitrogen doping facilitated by DAP and the activation process. CDAP800-350 also showed outstanding durability and excellent rate capability, which made it a promising candidate for supercapacitor applications in the future.

## 2. Experimental

### 2.1 Preparation of samples

Absorbent cotton was used as the starting material and diammonium hydrogen phosphate (DAP) of analytical grade was used as both the doping and activation reagent. The weight ratio of cotton and DAP was 1 : 1. Absorbent cotton (10 g) was immersed in DAP solution (10 g DAP dissolved in 250 mL DI water) for 2 hours at ambient temperature and was dried at 100 °C for 12 h. Then, the pretreated cotton and the control (cotton without pretreatment with DAP) were activated in a furnace at 800 °C under nitrogen atmosphere at a heating rate of 5 °C min^−1^ and were kept at 800 °C for 2 hours. After activation, the products were washed with DI water to remove impurities, dried at 100 °C for 5 h, and were designated as CDAP800 and C800.

Then CDAP800 samples were oxidized under air atmosphere for 5 h at different temperatures (300 °C, 350 °C and 400 °C), and these samples were designated as CDAP800-300, CDAP800-350 and CDAP800-400, respectively.

### 2.2 Material characterization

(1) X-ray diffraction (XRD) patterns were recorded on an XRD analyzer (D8-Advance, Bruker Advance X-ray diffractometer-AXS, Germany) equipped with a diffracted-beam monochromator using Cu Kα radiation (50 kV, 40 mA).

(2) The Raman spectroscopy (Horiba Scientific, France) was used to characterize the synthesized porous carbon samples with 532 nm excitation-beam wavelength.

(3) The morphologies and structures of the samples were observed by using a scanning electron microscopy (SEM) instrument (FE-SEM, ZEISS, SUPRA 55VP, Oberkochen, Germany) and transmission electron microscopy (TEM) instrument (H-600, Hitachi, Japan). Before TEM characterization, the samples were dispersed in ethanol and the suspensions were attached on a carbon-coated copper grid for measurements.

(4) Samples were degassed in vacuum at 200 °C for 3 h prior to the surface area analysis. Surface area was calculated by Brunauer–Emmet–Teller (BET) method from the adsorption–desorption branch of the isotherms (V-Sorb 2800P, China). The pore size distribution was analyzed by the BJH method.

(5) X-ray photoelectron spectroscopy (XPS) was recorded on an ESCALAB 250Xi (Thermo Fisher Scientific, America) scanning XPS microprobe with monochromatic Al Kα as the excitation source.

### 2.3 Electrochemical measurements

The electrochemical measurements were carried out on a CHI660E electrochemical workstation (Chenhua, Shanghai, China) at ambient temperature. A square platinum plate and Hg/HgO were used as the counter electrode and reference electrode, respectively. In a typical procedure, the electrodes were prepared by mixing the active material, carbon black, and polytetrafluoroethylene (PTFE) in a mass ratio of 80 : 10 : 10; all these were blended with ethanol, dropwise coated onto a titanium mesh (1 cm × 1 cm) under a pressure of 15 MPa, and then dried at 60 °C for 30 min in an oven to obtain electrodes. The loading mass of the active material on each electrode was 5.0 mg.

#### 2.3.1 Three-electrode testing

The electrochemical tests were measured using a three-electrode cell system with 6.0 M aqueous KOH solution as the electrolyte. Cyclic voltammetry (CV), galvanostatic charge–discharge (GCD) technique and electrochemical impedance spectroscopy (EIS) were important parameters in the electrochemical investigations. The potential window was set as −1 to 0 V. CV tests were conducted at different scan rates of 5, 10, 20, 50, 100 mV s^−1^, and GCD tests were conducted at a current density of 0.5, 1, 2, 5 and 10 A g^−1^. EIS was measured in the frequency range of 0.01 Hz to 0.1 MHz at the alternating current amplitude of 5.0 mV. The gravimetric specific capacitance was calculated with the three-electrode method according to eqn (1).

1 *C* = *I* × Δ*t*/*m* × Δ*V*

where *C* (F g^−1^) is the specific capacitance, *I* is the current density (A), Δ*t* (s) is the charge/discharge duration time, *M* is the overall mass loading of the active material in the coin device, and Δ*V* (v) is the potential window under the testing condition.

#### 2.3.2 Two-electrode testing

In a two-electrode system, the mass loading of the active material on each electrode in the symmetric supercapacitor was 5.0 mg, and the electrodes were prepared by mixing the active material, carbon black, and polytetrafluoroethylene (PTFE) in a mass ratio of 80 : 10 : 10; all these were blended with ethanol, coated onto a titanium mesh (1 cm × 1 cm) under a pressure of 15 MPa, and then dried at 60 °C for 30 min in an oven to obtain electrodes. The procedure is the same as that used for preparing the three-electrode system. The electrodes were made by the same above-mention method. A symmetrical supercapacitor was prepared from two similar-quality electrodes and separated by a filter paper membrane.

The electrochemical performance was tested in 6 M KOH aqueous solution. The specific capacitance was calculated according to eqn (2).

2 *C* = 2*I* × Δ*t*/*m* × Δ*V*

where *C* (F g^−1^) is the specific capacitance, *I* (A) is the discharge current, *m* (g) is the mass of materials, and Δ*V* (V) is the potential window.

The energy density (*E*, W h kg^−1^) and power density (*P*, W kg^−1^) of the two-electrode system were calculated by eqn (3) and (4), respectively.3
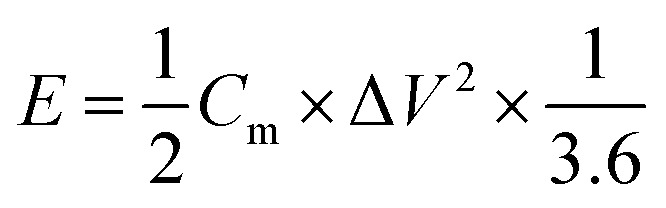
4
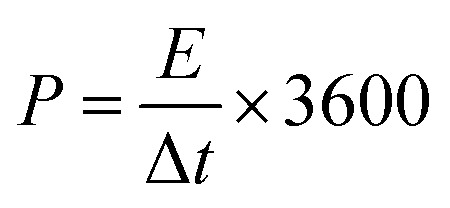
where *C*_m_ is the gravimetric capacitance on one electrode of the two-electrode symmetrical supercapacitor cell, *V* (V) is the voltage change within the discharge time, and Δ*t* (s) is discharge time.

## 3. Results and discussion

### 3.1 Structural and textural characterization

**Fig. 1:**
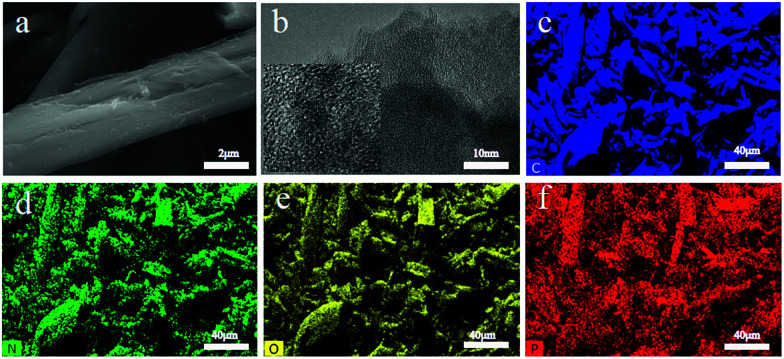
(a) SEM images, (b) TEM images, and EDS mapping images of CDAP800-350, and the corresponding EDX mapping of (c) C, (d) N, (e) O, and (f) P.

The morphology and structure were investigated by scanning electron microscopy (SEM) and transmission electron microscopy (TEM). Fig. 1(a) shows the typical morphology of CDAP800-350. The diameter of the carbon fiber is 8–10 μm. Fig. 1(b) is the microscopic structure of the CDAP800-350 sample obtained by TEM. The picture shows an amorphous structure with irregular domains, indicating some disorder defects and functional groups present in the samples.^23^

In order to probe the elemental distribution of the sample, EDS (energy-dispersive X-ray spectroscopy) mapping of carbon, oxygen, nitrogen, and phosphorus elements were performed and the results are shown in Fig. 1(c–f). All elements demonstrated a homogeneous distribution on the carbon sample surface, which indicated that O, P and N were successfully incorporated onto the surface of the prepared carbon materials.

**Fig. 2:**
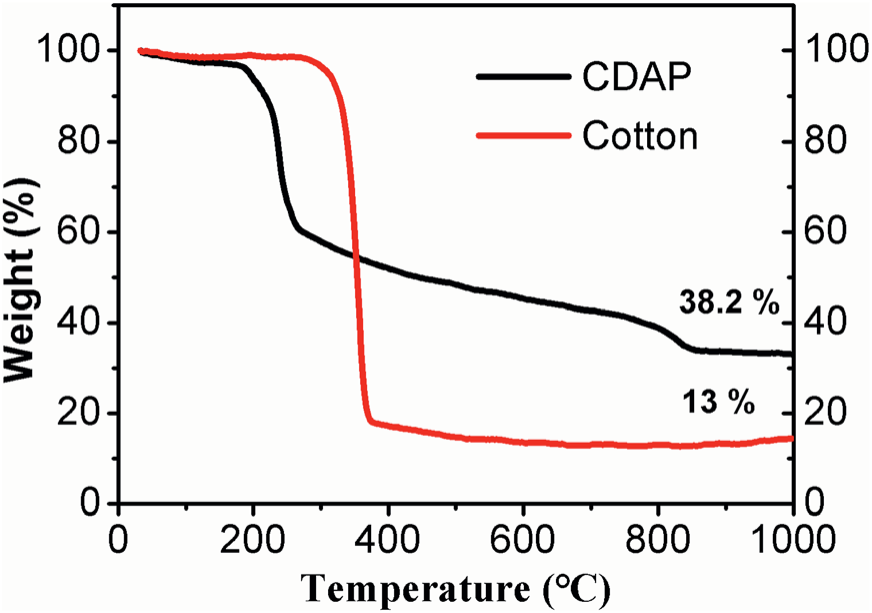
TGA curves of untreated cotton and cotton-treated with DAP (CDAP) fibers measured under nitrogen atmosphere.

Fig. 2 shows the TGA of cotton and CDAP; CDAP represents cotton immersed with DAP, and cotton represents the untreated cotton. From the figure, drastic thermal decomposition at 370 °C was observed for the cotton sample without DAP, and a solid residue occupied only 13.0 wt% of its initial mass at 800 °C. The low carbon yield of C800 was mainly due to levoglucose and tar formation. The more rapid the decomposition is, the larger the amount of volatiles produced.^24,25^

It was observed that cotton treated with DAP showed a large mass loss in the temperature range of 150–200 °C, which was attributed to the pyrolysis of cotton and DAP (to H_4_P_2_O_7_). However, compared to C800, the weight loss was significantly decreased after 270 °C. After the temperature increased beyond 800 °C, there was still a small mass loss, which was proposed to be due to the pyrolysis of P_2_O_5_.^24^ For the cotton treated with DAP, the solid residue retained 38.2 wt% of its initial mass at 800 °C. This value was significantly higher than for the sample without DAP treatment. This result suggested that DAP is an excellent impregnant to improve carbon yield. The possible explanation was that with pyrolysis of DAP, N and P were physically added or grafted to cotton, promoting phosphorylation and forming a more thermally stable amorphous charred residue containing N–P–O. The phosphorylation reactions occurred and accelerated the dehydration of cellulose, which was capable of altering the mode of cellulose decomposition and resulting in improved carbon yield.^26^

**Fig. 3:**
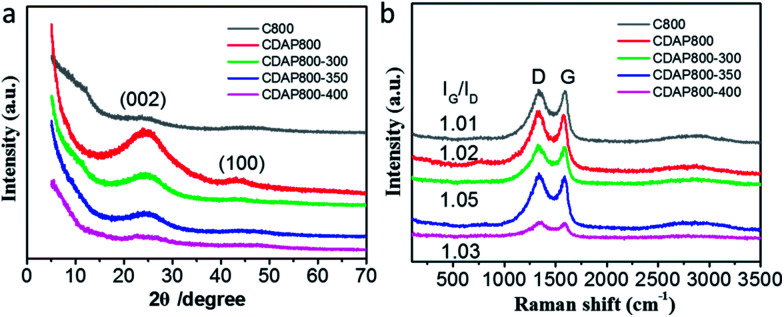
(a) XRD patterns, (b) Raman spectra of different carbon materials.

Wide-angle X-ray diffraction (XRD) was employed to characterize the crystallographic structures of the carbon materials. Fig. 3(a) showed two broad diffraction peaks around 25.0° and 43.5°, which were assigned to the (002) and (101) planes of graphitic carbon, respectively. The diffraction peak at 2*θ* = 20–30° broadened and weakened with elevated oxidation temperature. The position (angle) of the (002) band was believed to be related to the inter-lamellar spacing.^23^ The result implied that the CDAP800s had an amorphous structure with low graphitization degree. These smaller graphitic regions may be induced by the local lattice distortion with N and O doping. Another weak peak displayed at approximately 43.5° indicated the low degree of graphitization and high degree of interlayer condensation.^27^ With the oxidation temperature increasing during the oxidation process, the intensity of the peaks decreased. When oxidation temperature increased to 400 °C, the peak at 43.5° was just as small as that of C800. The result was probably caused by severe structural damage at elevated temperatures, which decreased the graphitization structure and electrical conductivity.

Raman spectra showed that all carbons exhibited two characteristic bands at around 1339 cm^−1^ (D band) and 1593 cm^−1^ (G band). D bands could be assigned as the disordered/defective structure of carbon, and G bands were associated with ordered graphitic structures due to the *E*_2g_ vibrational mode present in the sp^2^-hybridized carbon atoms in the graphitic layer.^27^ The degree of graphitization and damage was estimated using the intensity ratio of D/G band (*I*_D_/*I*_G_), which probably partially resulted from the decomposition of O- and N-containing functional groups at high temperature.^28^ As shown in Fig. 3(b), the D/G band (*I*_D_/*I*_G_) values of the oxidized samples were 1.01, 1.02, 1.05, and 1.03 for CDAP800, CDAP800-300, CDAP800-350, and CDAP800-400, respectively, implying these N- and O-doped porous carbons possessed disordered structures, especially CDAP800-350.

**Fig. 4:**
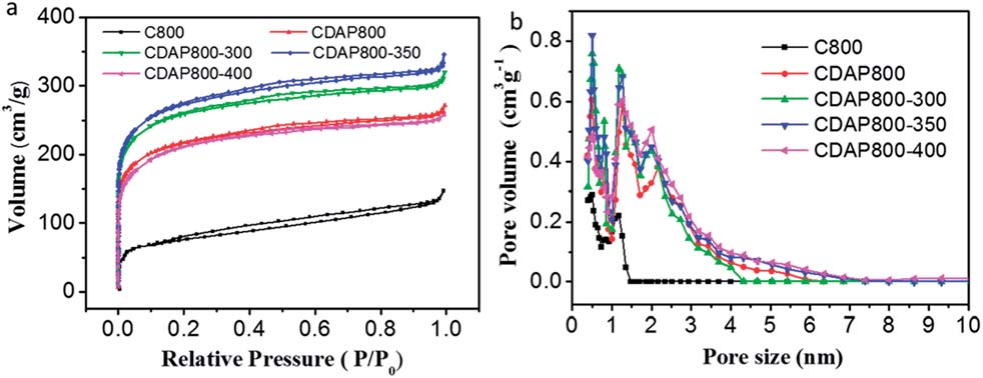
(a) N_2_ adsorption–desorption isotherms and, (b) pore size distributions of C800, CDAP800, CDAP800-300, CDAP800-350, and CDAP800-400 obtained by DFT method.

N_2_ adsorption–desorption measurements were conducted to evaluate the surface areas and pore structures of the as-prepared materials and the control sample. The N_2_ adsorption–desorption isotherms and pore size distribution are shown in Fig. 4. The isotherms were typical type I (typical of microporous carbons) with a pronounced N_2_ uptake at relatively low pressure, according to IUPAC, which implied the existence of substantial micropores.^29^ The pore size distribution of the samples is shown in Fig. 4(b). Results showed that the diameter of pore size distribution of all samples were in the range of 0.4 to 4 nm, which was consistent with the coexistence of micropores and mesopores.

The surface area and pore structure parameters of all samples are summarized in Table 1. The specific surface areas (*T*-plot) of C800, CDAP800, CDAP800-300, CDAP800-350, and CDAP800-400 samples were measured to be 268, 810, 967, 1022 and 772 m^2^ g^−1^, respectively, while the pore volumes of these samples were obtained as 0.228, 0.421, 0.495, 0.534 and 0.340 cm^3^ g^−1^, respectively. It was clear that the specific surface areas of the products obtained from DAP-pretreated cotton were much larger than from the control sample without pretreatment with DAP. From Table 1, it was found that majority of the pores were micropores, which was advantageous for improving specific capacitance because these pores could provide abundant active sites for ion storage.

**Table 1:** Surface and texture properties of C800 and CDAP800 samples^a^

Samples	*S*_BET_ (m^2^ g^−1^)	*S*_micro_ (m^2^ g^−1^)	*S*_meso_ (m^2^ g^−1^)	*V*_total_ (cm^3^ g^−1^)	*V*_micro_ (cm^3^ g^−1^)	*V*_meso_ (cm^3^ g^−1^)	DP (nm)
C800	268	160	108	0.228	0.077	0.151	2.0
CDAP800	810	731	79	0.421	0.315	0.106	2.1
CDAP800-300	967	887	80	0.495	0.382	0.113	2.1
CDAP800-350	1022	922	100	0.534	0.397	0.137	2.1
CDAP800-400	772	710	71	0.340	0.313	0.027	2.1

a *S*_BET_: BET surface area, *S*_micro_: surface area of micropores, *S*_meso_: surface area of mesopores, *V*_total_: total pore volume, *V*_micro_: pore volume of micropores, *V*_meso_: pore volume of mesopores, DP: average pore diameter.

However, mesopores could also be beneficial for penetration and transportation of electrolyte ions as well as because of their low resistance to ion transport.^18,30^ Results also demonstrated that different oxidation temperatures led to different in surface area and pore structure values even when using the same amount of DAP. The appropriate oxidation temperature could lead to the maximum surface area and pore volume. However, over oxidation at a very high temperature would actually destroy the carbon materials, which led to the destruction of its pore structure and reduction of the surface area. At high oxidation temperatures (400 °C), over oxidation could collapse the carbon framework. The collapse led to the blocking of some of the mesopores and micropores, resulting in a relatively low surface area and more amorphous carbon.^16,22^ These results suggested that the BET surface area of the as-prepared carbon materials was significantly influenced by both DAP treatment and oxidation temperature.

**Fig. 5:**
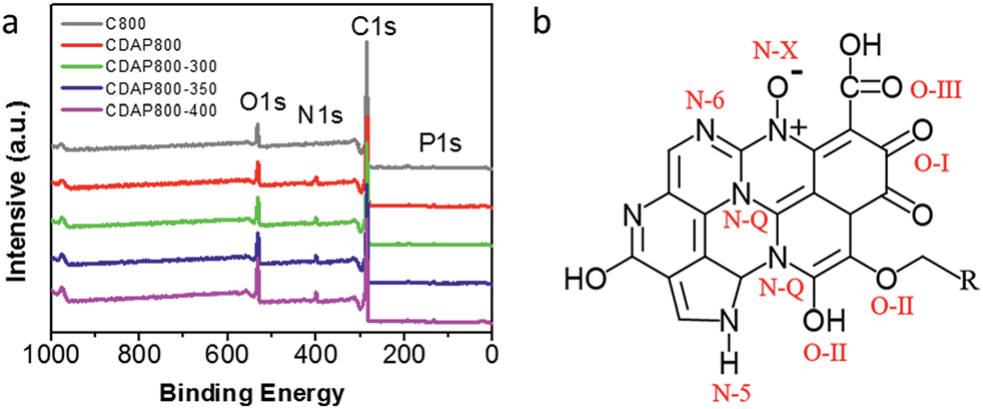
(a) XPS survey spectra of carbon samples, (b) schematic of different types of N and O in carbon material lattice.

As shown in Fig. 5(a), four characteristic peaks were observed at 299, 400, 531 and 145.2 eV, which corresponded to C 1s, N 1s, O 1s and P 1s. The chemical compositions are summarized in Table 2. All samples were found to have the same content of P, about 1.3%. Nevertheless, the content of N increased from 1.9% to 5.8% with appropriate activation and oxidation. It implied that the DAP treatment could increase the nitrogen content and acted as an effective doping reagent. At the same time, the oxygen content increased with increasing oxidation temperature. N and O groups mainly provided pseudocapacitance and O groups were beneficial for the wettability of the electrode.^31^

**Table 2:** Relative surface element content of different carbons measured by XPS

Samples	C%	N%	O%	P%
C800	88.1	1.9	8.6	1.3
CDAP800	83.8	5.5	9.4	1.3
CDAP800-300	82.3	5.6	10.9	1.3
CDAP800-350	81.1	5.8	11.8	1.3
CDAP800-400	76.6	6.2	15.8	1.3

**Fig. 6:**
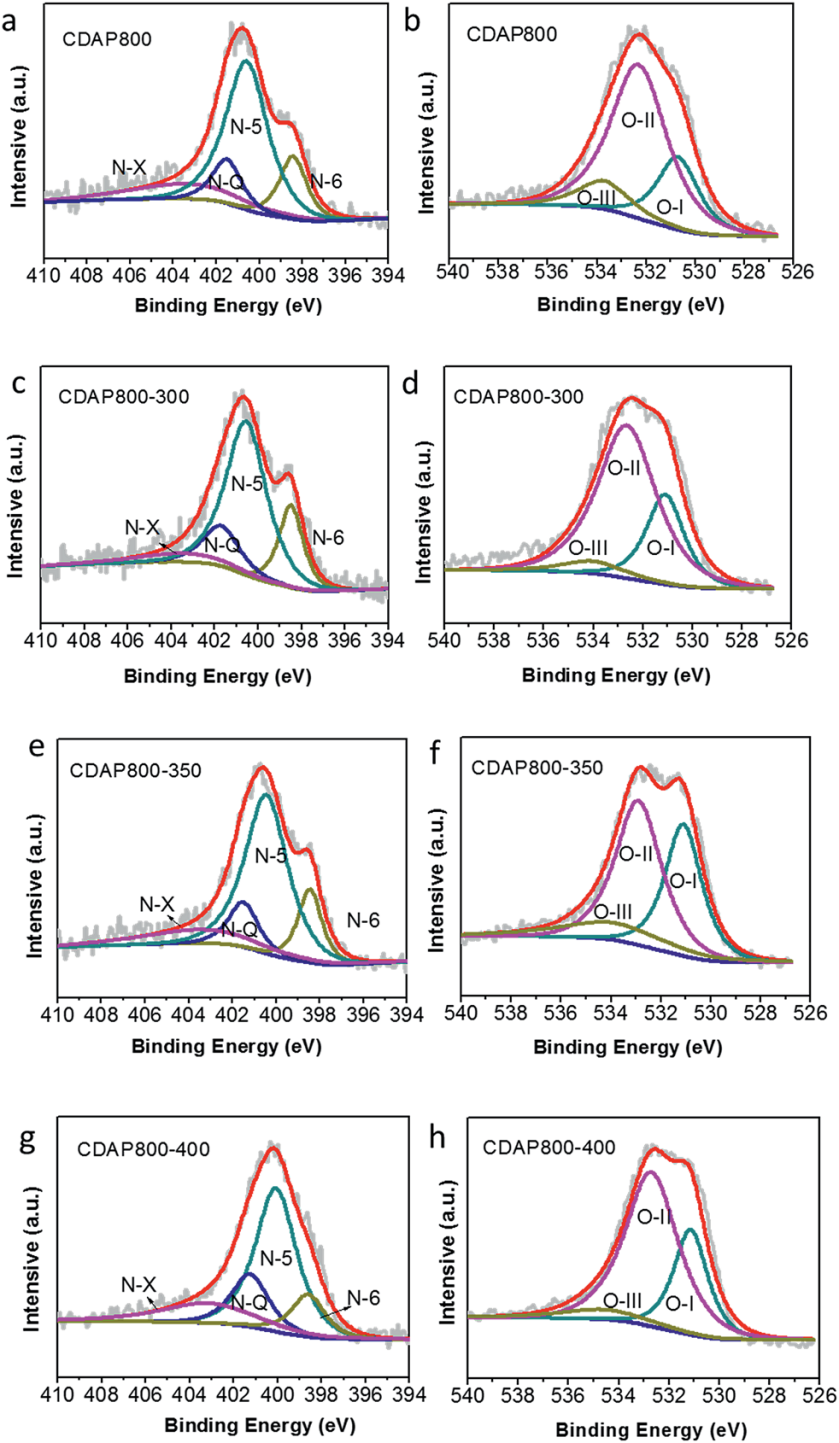
XPS spectra of the as-prepared carbon samples (a) N 1s of C800, (b) O 1s of C800, (c) N 1s of CDAP800-300, (d) O 1s of CDAP800-300, (e) N 1s of CDAP800-350, (f) O 1s of CDAP800-350, (g) N 1s of CDAP800-400, (h) O 1s of CDAP800-400.

High-resolution scans of N 1s of the samples are shown in Fig. 6(a, c, e and g). Nitrogen was fitted by four component peaks, 398.1, 400.2, 401.2 and 403.6 eV, which were ascribed to pyridine-N-6, pyrrole or pyridine-N-5, quaternary-N-Q and pyridine N-oxide (N-X), respectively. XPS high-resolution scans indicated that nitrogen atoms were inserted into the porous carbon skeleton at different binding states, as shown in Fig. 5(b). N-6 is sp^2^ N-bonded to two C atoms at the edge of the graphene layer and donates one p electron to the aromatic system.^32,33^ N-5 represents pyrrolic N in a five-membered ring that is associated with phenolic or carbonyl group on the neighboring carbon atom of the ring. N-Q is N bonded to three C atoms in the central or valley position of the graphene layer, and N–O is N bonded to O at the edges of graphene layer. In Table 3, N-5, N-6 and N-Q N accounted for a large proportion (over 80%) of N content in all samples. Generally speaking, the presence of N-6 and N-5 could enhance the pseudocapacitance by faradaic redox reactions, while intercalated N-Q between graphene sheets could facilitate the electron transfer in the carbon skeleton, enhancing the electronic conductivity of carbon to ensure rapid and reversible ion/charge transfer and exchange.^34–36^ It was noteworthy that the CDAP800-350 sample had the largest proportion of the above-mentioned types of N bonding (90%).

**Table 3:** Relative N and O contents determined by high-resolution N 1s and O 1s XPS spectra

Samples	N-6	N-5	N-Q	N-X	O*-*I	O-II	O-III
CDAP800	15.7	56.8	11.8	15.7	23.1	65.7	11.2
CDAP800-300	17.2	57.2	14.1	11.5	29.2	61.3	9.4
CDAP800-350	15.4	58.9	19.4	13.8	36.3	50	13.7
CDAP800-400	12.8	53.3	17.7	16.2	28.3	65	6.7

In Table 3, it was found that with increasing oxidation temperature, the oxygen content increased accordingly. Oxygen greatly contributed to wettability of the carbon surface and specific capacitance. But very high oxygen content decreased the electrical conductivity, which was unfavorable for stability of the electrode at a high current density. XPS high-resolution scans of O 1s are shown in Fig. 6(b, d, f and h). Three different O states were observed, *i.e.*, C=O oxygen in carbonyl- or quinone-type groups (O-I), C–OH phenol groups/C–O–C ether groups (O-II) and O=C–O (chemisorbed oxygen, carboxylic groups)/or water (O-III), which corresponded to peaks at 531.3, 532.7 and 534.2 eV, respectively. O in different chemical states within the carbon matrices are schematically shown in Fig. 5(b).^37^ O-II is a type of O bonding where hydrogen bonds interact with a water molecule. O-II is beneficial to the surface wettability of the carbon materials. All samples had large amount of O-II, which indicated that they showed low resistance to ion transport between the electrolyte and material surface. It should be pointed out that only the quinine-type oxygen (O-I) was electrochemically active, which was favorable for improving pseudocapacitance of the electrode.^30^ CDAP800-350 and CDAP800-400 all showed high oxygen content, especially quinine-type oxygen, which may be one of the reasons why they performed well in the electrochemical measurements.

### 3.2 Electrochemical properties

**Fig. 7:**
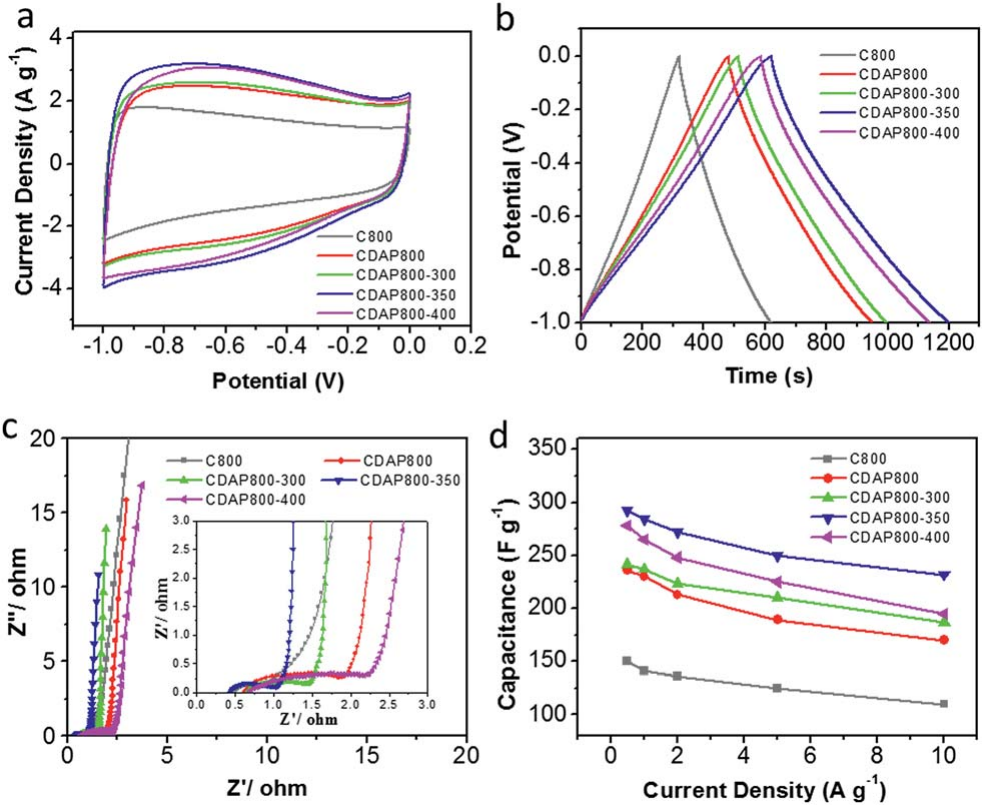
Electrochemical performance in a three-electrode system with 6 M KOH as the electrolyte. (a) CV curves at 10 mV s^−1^, (b) galvanostatic charge/discharge profiles at a current density of 0.5 A g^−1^, (c) Nyquist plots. (d) Specific capacitance at different current densities.

The electrochemical properties of the as-prepared carbon were evaluated as electrode materials in a three-electrode system using 6 M KOH as the electrolyte. The cyclic voltammograms (CVs) are shown in Fig. 7(a) at a scan rate of 10 mV s^−1^. The proportion to the area of the CV curve is related to the capacitance of an electrode; generally, the larger the quasi-rectangular area of the CV curve, the higher the electrochemical capacitance. The CDAP800-350 had the largest proportion to the area of its CV curve; thus, it had larger electrochemical capacitance than other samples at the same scanning rate and in the same potential window. CV curves of all samples had a quasi-rectangular shape, which indicated that electrical energy was mainly stored in the electrical double layer.^38^ The redox peaks were found at 0.6 V for all samples, which were attributed to faradaic pseudocapacitance resulting from redox reactions due to the O (hydroxyl, quinone, carboxyl and lactone groups) and N (pyridinic and pyrrolic groups) functionalities.^39^

Fig. 7(b) shows the galvanostatic charge/discharge (GCD) curves at a current density of 0.5 A g^−1^. The GCD curves of all samples were found to have a quasi-isosceles triangular shape between 0 and −1 V *versus* the Hg/HgO electrode. The charge–discharge curves of the electrodes were not straight lines but showed distinct distortion. This phenomenon indicated that double-layer capacitance was the major contributor to the performance and pseudocapacitance also existed.^40,41^

From GCD results, the CDAP800-350 exhibited the highest capacitance of 292 F g^−1^ at a current density of 0.5 A g^−1^, which was higher than that of C800 (160 F g^−1^), CDAP800-300 (240 F g^−1^) and CDAP800-400 (267 F g^−1^). The CV curves showed the same trend as well. CDAP800-350 had the largest specific capacitance. The specific capacitance was determined by both surface area and surface element content. CDAP800-350 had the largest specific surface area (1022 m^2^ g^−1^) and largest total pore volume (0.534 cm^3^ g^−1^), which offered abundant adsorbing sites for ions. CDAP800-350 also was found to have plenty of micropores and mesopores, which provided helpful transportation channels for the electrolyte to diffuse into the micropores. In addition, O and N content also helped improve the specific capacitance. O content was helpful in increasing electrode surface wettability as well as in generating more electrochemical active sites, but too much O decreased electron flow rate and increase charge-transfer resistance. This explained why CDAP800-400 had lower surface area and pore volume than CDAP800-300, but had larger specific capacitance.

Electrochemical impedance spectroscopy (EIS) was carried out with an alternating current voltage amplitude of 5 mV in a frequency range of 0.01 Hz to 100 kHz. EIS provided complementary information to further understand the capacitive behaviors of the samples, as shown in Fig. 7(c). At low frequencies (Warburg diffusion component), the slope of the impedance plots was nearly a vertical line, indicating the electrolyte ions could easily access the surface without diffusion limitations. The tail with a slope of 45° in the intermediate frequency region represented the Warburg diffusion resistance (*Z*_w_), reflecting the characteristics of ion diffusion into the active materials. The shorter the length of this diffusion section in capacitors, the lower the ion diffusion resistance, which was mainly attributed to the enhanced surface hydrophilicity by increasing O content.^36,42^ At high frequency, the first intersection point on the *Z*-axis corresponded to the solution resistance (*R*_s_) and the semicircle intercepts on the Nyquist plot corresponded to the charge-transfer resistance (*R*_ct_) of the electrode materials, derived from the reversible faradaic reactions of the oxygen-containing groups.^17^

Furthermore, the stability at high current density is an important factor for the application of a supercapacitor. Discharge capacitances at various current densities were calculated from the galvanostatic charge–discharge curves from 0.5 A g^−1^ to 10 A g^−1^ in Fig. 7(d). It was concluded that the calculated specific capacitance of CDAP800-350 was higher than that of other samples at all tested current densities. The specific capacitances of the CDAP800-350 electrode were measured to be 292, 284, 272, 250, and 233 F g^−1^ at current densities of 0.5, 1, 2, 5 and 10 A g^−1^, respectively. A rate capability of 80% retention was achieved, which was much higher than that of other samples, suggesting good stability at high current density.

Remarkably, all samples showed small *R*_s_ values, low Warburg diffusion resistance (*Z*_w_) and large straight slopes. CDAP800-350 exhibited the best rate performance due to its high surface area, special pore size distribution and moderate O content. CDAP800-350 electrode presented the lowest *R*_ct_ among all the electrodes, suggesting its superior rate capability. Thus, pretreatment with DAP and appropriate oxidation temperature was indeed of paramount importance for improving the capacitive performance of cellulose-derived carbon.

**Fig. 8:**
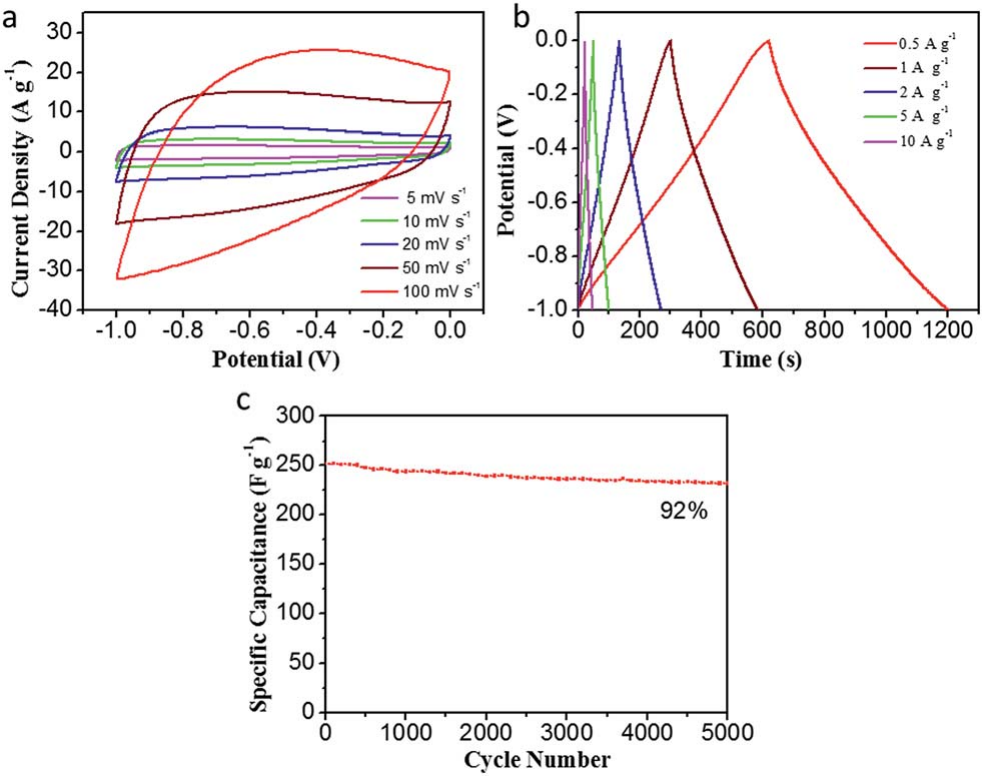
Electrochemical performance of CDAP800-350 in a three-electrode system. (a) CV curves at various scan rates of 5–100 mV s^−1^, (b) GCD curves tested at 0.5–10 A g^−1^, (c) capacitance retention of the as-prepared samples over 5000 cycles at 5 A g^−1^.

The electrochemical performance of CDAP800-350 in a three-electrode system is presented in Fig. 8. Fig. 8(a) shows a quasi-rectangular shape at different scan rates (5–100 mV s^−1^), indicating good capacitive behavior of the electrode and fast ion-transport into the electrochemically active surface. From GCD curves of the CDAP800-350 electrode in Fig. 8(b), it was found that the GCD curves were not straight lines but showed slight distortion, suggesting their combined capacitance originates from double-layer capacitance and faradaic pseudocapacitance, which is consistent with the CV results.^12,43^ The evaluation of cycle stability for CDAP800-350 was conducted at a current density of 5 A g^−1^. Results in Fig. 8(c) show that after 5000 cycles, the specific supercapacitance of CDAP800-350 showed a moderate decrease from 250 F g^−1^ to 231 F g^−1^. About 92% of the initial specific capacitance was retained.

**Fig. 9:**
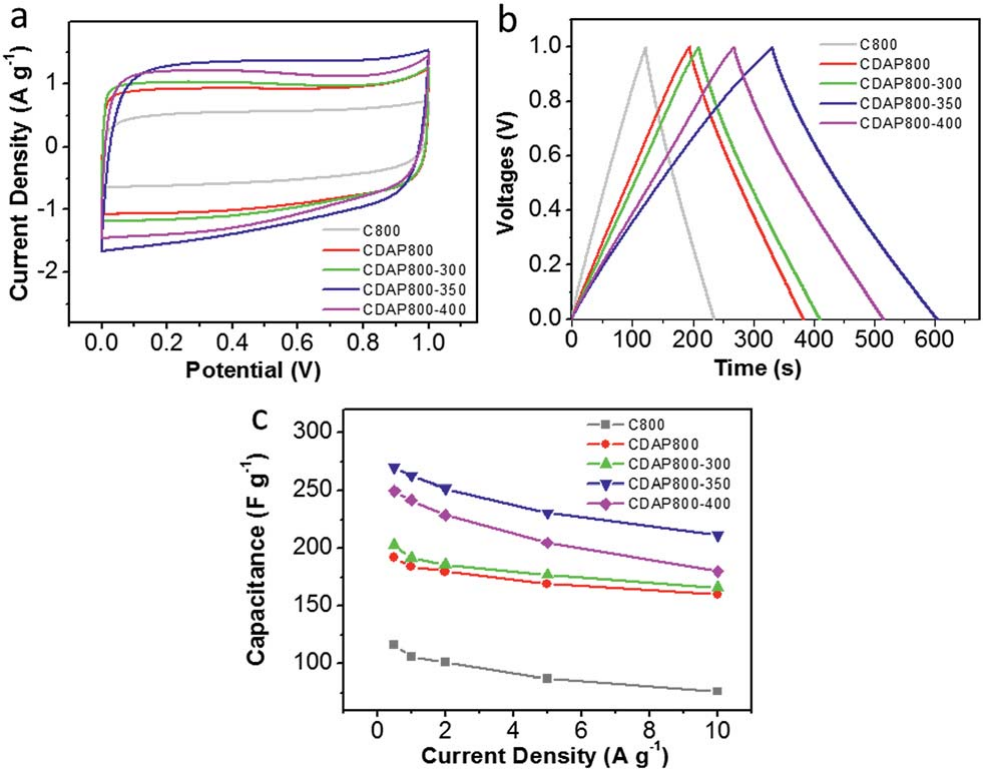
Electrochemical performance in a two-electrode system with 6 M KOH as the electrolyte. (a) CV curves at 10 mV s^−1^, (b) galvanostatic charge/discharge profiles at a current density of 0.5 A g^−1^, (c) specific capacitance at different current densities.

A two-electrode configuration was used with 6 M KOH electrolyte to evaluate the use of the samples as electrodes of a symmetric supercapacitor. The carbon materials were packaged as symmetric devices with two identical electrodes and were tested with CV and GCD experiments. Fig. 9(a) shows typical square-shaped CV curves for both carbon electrodes at 10 mV s^−1^, indicating good double-layer capacitance. The GCD plots of C800 and CDAP800 samples showed an isosceles-triangle shape at a current density of 0.5 A g^−1^ (Fig. 9(b)), implying good charge and discharge reversibility.

Discharge capacitances at various current densities were calculated from the galvanostatic charge–discharge curves from 0.5 A g^−1^ to 10 A g^−1^ in Fig. 9(c). Corresponding specific capacitances followed the order CDAP800-350 (270 F g^−1^ at 0.5 A g^−1^) > CDAP800-400 (250 F g^−1^ at 0.5 A g^−1^) > CDAP800-300 (203 F g^−1^ at 0.5 A g^−1^) > CDAP800 (192 F g^−1^ at 0.5 A g^−1^) > C800 (116 F g^−1^ at 0.5 A g^−1^).

**Fig. 10:**
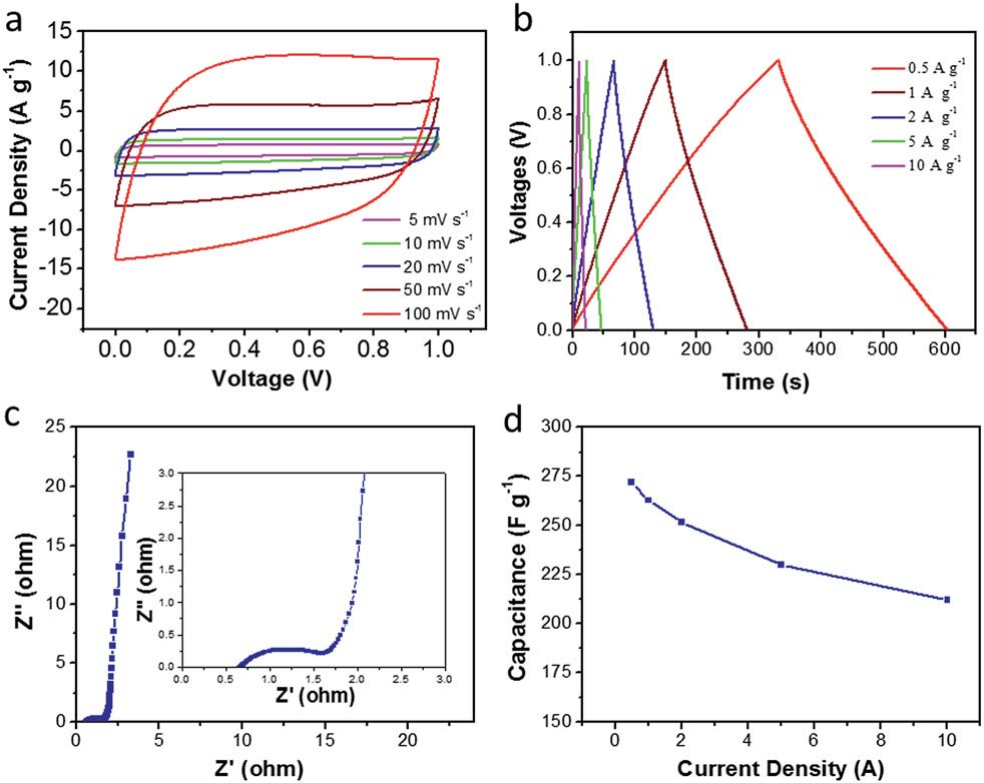
Electrochemical performance of CDAP800-350 in a two-electrode system. (a) CV curves at various scan rates of 5–100 mV s^−1^, (b) GCD curves tested at 0.5–10 A g^−1^, (c) Nyquist plots, (d) specific capacitance at different current densities.

In order to further evaluate the supercapacitor performance for energy storage, a two-electrode symmetric supercapacitor based on the CDAP800-350 electrode was tested in 6 M KOH electrolyte. The typical CVs of the symmetric supercapacitor at different scan rates from 5 to 100 mV s^−1^ are displayed in Fig. 10(a). The CVs of CDAP800-350 showed a rectangular shape with a scan rate ranging from 5 mV s^−1^ to 100 mV s^−1^. The shape of the CVs did not change significantly even at a high scan rate of 100 mV s^−1^, implying good double-layer capacitance, decent rate capability and rapid ion transportation and excellent rate capability.^5,22,29^ The GCD curves in Fig. 10(b) show a regular symmetric triangular shape, suggesting good electrochemical charge and discharge reversibility, and the corresponding electrode specific capacitance of the symmetric supercapacitor was calculated from the GCD curves.

Results showed that the CDAP800-350-based symmetric supercapacitor achieved a high specific capacitance of 270 F g^−1^ at a current density of 0.5 A g^−1^. Impressively, as shown in Table 4, under the same testing condition, CDAP800-350 also achieved higher gravimetric capacitances compared to similar carbon materials reported in the literature.^11,14,21,27,28,44,45^Fig. 10(c) shows a Nyquist plot of the symmetric cell of the CDAP800-350-based supercapacitor. The small semicircle in the high-frequency region indicated a low transfer resistance (*R*_ct_) caused by faradaic reactions. The vertical part of the curve in the low-frequency region was ascribed to the standard electronic double-layer capacitor (*C*_dl_) at the interface between the electrode and electrolyte. Furthermore, the specific capacitances of CDAP800-350 at different current densities are shown in Fig. 10(d). The specific capacitance was measured to be 270 F g^−1^ at a current density of 0.5 A g^−1^ and 212 F g^−1^ at 10 A g^−1^, implying a rate capability of 79% retention.

**Table 4:** Comparison of specific capacitances of porous carbon materials in a two-electrode cell with 6 M KOH solution as the electrolyte

Entry	Materials	Current density (A g^−1^)	*C*_g_ (F g^−1^)	Ref.
1	N-Doped graphene (GN)	0.5	185	28
2	Wood sawdust-derived carbon fiber	0.5	225	11
3	Hydrogel-derived porous carbon	0.5	214	44
4	O- and N-doped carbon nanosheets	0.5	220	21
5	Pine-needle-derived N doped carbon	0.1	236	27
6	Nitrogen-doped hierarchical porous carbon	0.1	270	45
7	N-Doped porous carbon materials	0.5	289	14
8	Cotton-derived N-doped carbon	0.5	270	This work

**Fig. 11:**
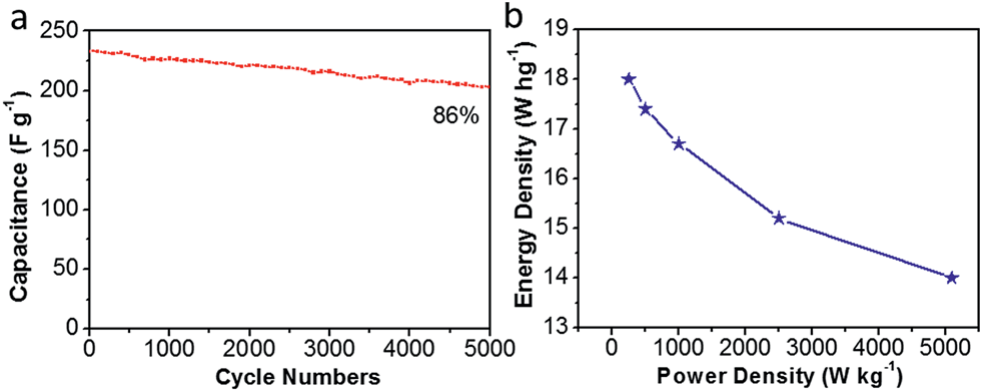
Electrochemical performance of CDAP800-350 in a two-electrode system. (a) Capacitance retention of the as-prepared carbon materials over 5000 cycles at 5 A g^−1^, (b) energy density *vs.* power density curve.

Moreover, Fig. 11(a) shows the long-term cycling stability of the as-assembled supercapacitors. The cycle stability of CDAP800-350 is about 86% of the initial specific capacitance after 5000 charge/discharge cycles at a high current density of 5.0 A g^−1^. More importantly, volumetric Ragone plots (Fig. 11(b)) of the assembled symmetric cell displayed a high energy density of 18 W h kg^−1^ at a power density of 250 W kg^−1^.

## 4. Conclusions

In summary, a facile and effective route to prepare N- and O-doped porous carbon from cotton was developed for supercapacitor electrode materials. The obtained CDAP800-350 electrode material exhibited a high surface area of 1022 m^2^ g^−1^ and a high oxygen (11.8 wt%) and nitrogen (5.8 wt%) content, which played an important role in fast ion transport and good electrical conductivity as well as good charge storage. Moreover, the CDAP800-350 electrode exhibited excellent electrochemical performance with a maximum specific capacitance of 292 F g^−1^ in 6 M KOH aqueous electrolyte. In a two-electrode symmetric supercapacitor, the CDAP800-350 also showed a high capacitance of 270 F g^−1^ at 0.5 A g^−1^ and good cycling stability (89% retention at a current density of 5 A g^−1^) after 5000 charge–discharge cycles. It also achieved a high energy density of 18 W h kg^−1^ at a power density of 250 W kg^−1^.

This facile biomass based approach provides an alternative solution for preparing O- and N-doped porous carbon that could be a promising candidate for supercapacitor applications in the future.

## Conflicts of interest

There are no conflicts to declare.

## Supplementary Material
